# Cholinergic agonists reduce blood pressure in a mouse model of systemic lupus erythematosus

**DOI:** 10.14814/phy2.13213

**Published:** 2017-04-10

**Authors:** Amber S. Fairley, Keisa W. Mathis

**Affiliations:** ^1^ Institute for Cardiovascular and Metabolic DiseasesUniversity of North Texas Health Science CenterFort WorthTexas

**Keywords:** Cholinergic anti‐inflammatory pathway, hypertension, inflammation, kidney, nicotine, renal injury

## Abstract

Increased inflammation arising from an abnormal immune response can damage healthy tissue and lead to disease progression. An important example of this is the accumulation of inflammatory mediators in the kidney, which can subsequently lead to hypertension and renal injury. The origin of this inflammation may involve neuro‐immune interactions. For example, the novel vagus nerve‐to‐spleen mechanism known as the “cholinergic anti‐inflammatory pathway” controls inflammation upon stimulation. However, if this pathway is dysfunctional, inflammation becomes less regulated and chronic inflammatory diseases such as hypertension may develop. Systemic lupus erythematosus (SLE) is an autoimmune disease with aberrant immune function, increased renal inflammation, and prevalent hypertension. We hypothesized that the cholinergic anti‐inflammatory pathway is impaired in SLE and that stimulation of this pathway would protect from the progression of hypertension in SLE mice. Female SLE (*NZBWF1*) and control (*NZW*) mice were administered nicotine or vehicle for 7 days (2 mg/kg/day, subcutaneously) in order to stimulate the cholinergic anti‐inflammatory pathway at the level of the splenic nicotinic acetylcholine receptor (*α*7‐nAChR). Blood pressure was assessed posttreatment. Nicotine‐treated SLE mice did not develop hypertension and this lower blood pressure (compared to saline‐treated SLE mice) coincided with lower splenic and renal cortical expression of pro‐inflammatory cytokines. These data provide evidence that the cholinergic anti‐inflammatory pathway is impaired in SLE. In addition, these data suggest that stimulation of the cholinergic anti‐inflammatory pathway can protect the kidney by dampening inflammation and therefore prevent the progression of hypertension in the setting of SLE.

## Introduction

More than 25% of the world's adult population was hypertensive in 2000 and the prevalence of hypertension is projected to increase by 29% by the year 2025 (Kearney et al. 2005). Antihypertensive therapies have been developed with the objective of decreasing blood pressure; however, the ultimate goal is to prevent the cardiovascular and renal complications associated with elevated blood pressure (Staessen et al. 2003). It is critical to understand the pathophysiology of hypertension and to identify the key factors in disease progression and maintenance in order to determine novel therapeutic targets.

Chronic inflammation, specifically in the kidney, has recently been associated with the progression of hypertension (Miguel et al. 2010; Heijnen et al. 2013; McMaster et al. 2015; Xiao et al. 2015). Immunosuppressive therapy and/or blockade of inflammatory mediators have been shown to reduce inflammation in the kidneys and protect from hypertension in both humans and animals (Rodriguez‐Iturbe et al. 2001; Herrera et al. 2006; Venegas‐Pont et al. 2010); therefore, it is of interest to determine the factors that play a role in the development of renal inflammation in the hypertensive host. Recent evidence suggests that the immune system and nervous system act together to regulate inflammation (Steinman 2004; Tracey 2009; Sundman and Olofsson 2014). One interaction of interest is the vagally‐mediated “cholinergic anti‐inflammatory pathway.” The cholinergic anti‐inflammatory pathway consists of neurotransmission from the vagus nerve to the splenic nerve, which promotes the release of splenic norepinephrine (Tracey 2002). Once norepinephrine binds its adrenergic receptor on splenic T cells, these cells release acetylcholine, which in turn, activates the alpha 7 subunit of the nicotinic acetylcholine receptor (*α*7‐nAChR) on other splenic immune cells (Wang et al. 2003; Rosas‐Ballina et al. 2011). Studies have shown that stimulation of this pathway ultimately results in a blunted release of pro‐inflammatory cytokines like tumor necrosis factor (TNF)‐*α* from the spleen, and an overall suppression of systemic inflammation and tissue injury (Borovikova et al. 2000a,b; Dowling et al. 2007; Rosas‐Ballina et al. 2008; Nizri et al. 2009; Van Maanen et al. 2009; Leib et al. 2011; Li et al. 2011). Whether an impaired cholinergic anti‐inflammatory pathway contributes to the heightened splenic and therefore systemic (i.e., renal) inflammation and whether this increased renal inflammation promotes increases in blood pressure is not known.

Recent studies have demonstrated the importance of the cholinergic anti‐inflammatory pathway in the regulation of pro‐inflammatory mediators in chronic inflammatory disorders such as sepsis, rheumatoid arthritis, and multiple sclerosis (Wang et al. 2004; Goldstein et al. 2007; Nizri et al. 2009) Systemic lupus erythematosus (SLE) is a chronic autoimmune disease characterized by renal inflammation, renal injury and prevalent hypertension (Budman and Steinberg 1976). In addition, patients with SLE can present with autonomic neuropathy (Liote and Osterland 1994; Maule et al. 1997; Louthrenoo et al. 1999; Stojanovich et al. 2007), which would suggest a hypoactive vagus nerve and potentially an impaired cholinergic anti‐inflammatory pathway. We hypothesized that the cholinergic anti‐inflammatory pathway is impaired in SLE and that stimulation of the pathway could provide protection from the exacerbated release of inflammatory mediators from the spleen, the development of systemic (renal) inflammation, and hence hypertension. In this study, we investigated whether activation of the cholinergic anti‐inflammatory pathway at the level of the *α*7‐nAChR attenuates the development of hypertension in a well‐established genetic mouse model of SLE. In addition, we investigated the influence of this pathway on the regulation of renal inflammation in SLE mice. We demonstrated that the cholinergic agonist, nicotine, is capable of altering splenic and renal inflammation in SLE mice and ultimately protects from the development of hypertension and renal injury.

## Materials and Methods

### Animals

Female SLE (*NZBWF1/J*) and control (NZW/LacJ) mice were obtained from Jackson Laboratories (Bar Harbor, ME) at 5–6 weeks of age. All mice were maintained on a 12‐h light/dark cycle in temperature‐controlled rooms with access to food and water ad libitum. Body weight measurements were recorded weekly starting at 30 weeks of age. All animal studies were approved by the University of Mississippi Medical Center Institutional Animal Care and Use Committee (IACUC) and were in accordance with National Institutes of Health (NIH) Guide for the Care and Use of Laboratory Animals.

### Nicotine administration

At 33 weeks of age, animals were divided into four groups: control/saline, control/nicotine, SLE/saline, SLE/nicotine. Mini‐osmotic pumps were surgically implanted into SLE and control mice. Animals were infused subcutaneously with nicotine hydrogen tartrate salt (Sigma, St. Louis, MO; 2 mg/kg/day) or saline for 7 days. The dose of nicotine was determined from previous studies that suggest that 2.0 mg/kg of nicotine infused subcutaneously suppresses pro‐inflammatory mediators in mice (Nizri et al. 2009). Plasma cotinine was measured by ELISA (Calbiotech, Spring Valley, CA) to confirm the presence of nicotine in the treated mice at the time of experiment. In order to separate the effects of nicotine on ganglionic and peripheral cholinergic receptors, a selective *α*7‐nAChR agonist, PNU‐282987 (0.38 mg/kg/day, IP) or vehicle (4% DMSO in saline) was administered for 28 consecutive days in a different subset of animals. The dose of PNU‐282987 was determined from previous studies that suggests that 0.38 mg/kg of the agonist administered intraperitoneally suppresses pro‐inflammatory mediators in rodents (Li et al. 2011).

### Blood pressure measurements

At 34 weeks of age (following 1 week of nicotine infusion), catheters were implanted into the left carotid artery as previously described (Mathis et al. 2011, 2012, 2013). Mean arterial pressure was then measured in conscious mice via pressure transducers for 1.5 h for two consecutive days post‐surgery. At the end of the study, all animals were perfused and euthanized. Tissues (i.e., kidneys and spleen) were harvested and stored for biochemical analysis.

### Renal injury

Animals were placed in metabolic cages for approximately 24 h in order to collect urine for analysis of urinary albumin, an index of renal injury. Urinary albumin was measured via dipstick (Siemens) and semiquantitatively assigned “grades” on a scale of 0–4 as follows: 0, trace; 0.5, trace‐30 mg/dL; 1, 30 mg/dL; 1.5, 30–100 mg/dL; 2, 100 mg/dL; 2.5, 100–300 mg/dL; 3, 300 mg/dL; 3.5, 300–2000 mg/dL; 4, 2000+ mg/dL. Albuminuria (at 34 weeks) was confirmed via ELISA (Alpha Diagnostics, San Antonio, TX). Data are presented as albumin excretion rate, which was calculated based on the concentration and volume of urine collected over time.

Paraffin‐embedded kidneys were stained with periodic acid‐Schiff (PAS) to assess glomerulosclerosis index as previously described (Mathis et al. 2014).

### Inflammatory mediators

The renal cortex was dissected from the right kidney of each mouse and the spleen, renal cortex, and renal medulla were homogenized at 4°C in RIPA buffer. Western blots were performed using splenic, renal cortical, and renal medullary homogenates as previously described (Mathis et al. 2012, 2013, 2014, 2014). Tumor necrosis factor (TNF)‐*α* and monocyte chemoattractant protein (MCP)‐1 were used as markers of inflammation, whereas interleukin (IL)‐10 was used as a marker of anti‐inflammation. TNF‐*α* was detected using a mouse monoclonal anti‐TNF‐*α* (1:250; Santa Cruz, Dallas, TX; 26 kDa). MCP‐1 was detected using a rabbit polyclonal anti‐MCP‐1 (1:2000; Abcam, Cambridge, MA; dimerized at ~37 kDa). IL‐10 was detecting using a mouse monoclonal anti‐IL10 (1:200; Santa Cruz, Dallas, TX; 20 kDa). Proteins were visualized using an HRP‐conjugated donkey anti‐mouse IgG (1:10000; Rockland, Limerick, PA), donkey anti‐rabbit IgG (1:1000; Rockland), or goat anti‐mouse (1:5000; Rockland), respectively. All Western blots were imaged and analyzed using the ChemiDoc MP Imaging System and ImageLab Software Version 5.1 (Bio‐Rad Laboratories, Inc., Hercules, CA). Western blot data are presented as a ratio of densitometry units of protein, based on band optical density, and normalized to total protein.

### Statistical analysis

All data are calculated as mean ± standard error of the mean (SEM) and statistical analyses were performed using SigmaPlot 11.0 (Systat, Richmond, CA). Statistical differences between multiple groups were determined by two‐way ANOVA followed by the Holm‐Sidak method.

## Results

### Subcutaneous nicotine suppresses splenic inflammation in SLE mice

**Figure 1:**
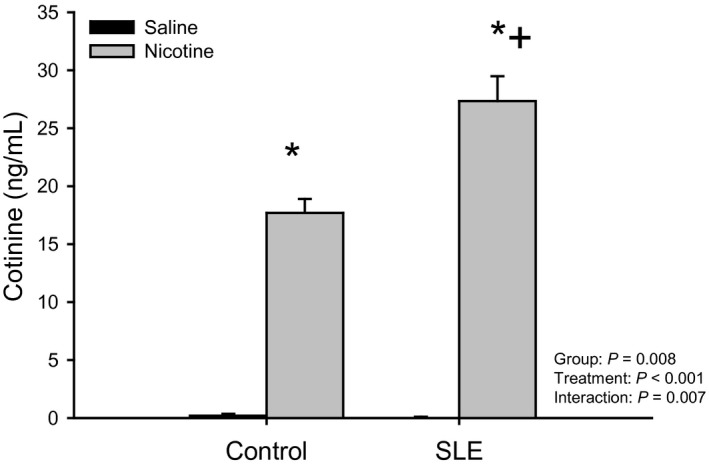
Chronic nicotine infusion increases plasma cotinine levels: Plasma cotinine (ng/mL) assessed by ELISA in control and SLE mice administered saline or nicotine for 7 days. *n *=* *3–4 plasma samples/group; P* versus corresponding saline‐treated; *P* + versus Control/Nicotine.

Plasma cotinine (ng/mL; Fig. 1) was increased in both control (17.70 ± 1.20 vs. 0.2 ± 0.2; *P* < 0.001) and SLE mice (27.33 ± 2.15 vs. 0.07 ± 0.03; *P* < 0.001) treated with nicotine (as compared to saline‐treated mice). SLE/nicotine mice had higher plasma cotinine than control/nicotine mice (*P* < 0.001). These data confirm the presence of nicotine in the treated mice at the time of experiment.

**Figure 2:**
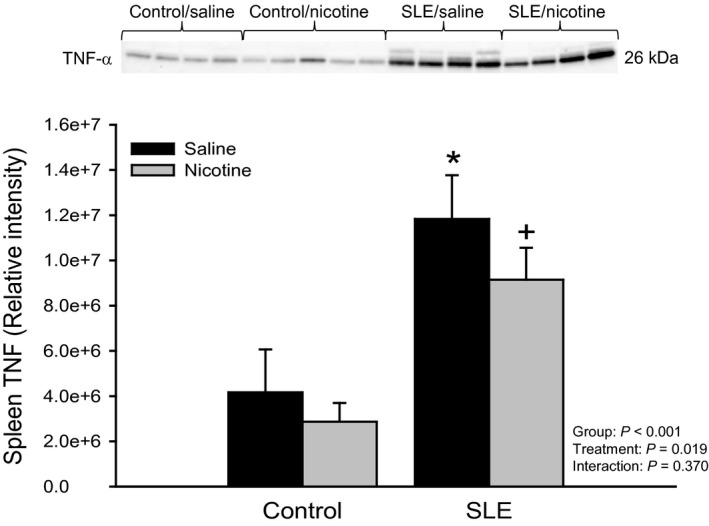
Chronic nicotine exposure blunts splenic TNF‐*α* in female SLE mice: The release of a splenic inflammatory mediator was measured in order to confirm stimulation of the cholinergic anti‐inflammatory pathway at the level of the *α*7‐nAChR. Protein expression of TNF‐*α* assessed by Western blot in spleens of control and SLE mice administered saline or nicotine for 7 days. Data are normalized to total protein. *n *=* *4–5 spleens/group; P* versus Control/Saline; *P* + versus SLE/Saline. TNF, tumor necrosis factor.

Splenic pro‐inflammatory cytokine production has been shown to be attenuated upon stimulation of the cholinergic anti‐inflammatory pathway (Borovikova et al. 2000b; Rosas‐Ballina et al. 2011). In the current study, we activated the cholinergic anti‐inflammatory pathway at the level of the splenic *α*7‐nAChR with the use of subcutaneous nicotine and measured TNF‐*α* in the spleen as proof of concept. Splenic TNF‐*α* expression (normalized to total protein) was increased in SLE mice compared to controls (1.2e7 ± 9.7e5 vs. 4.2e6 ± 9.5e5; *P* < 0.001; Fig. 2). SLE mice treated with nicotine had significantly lower TNF‐*α* (9.1e6 ± 7.1e5; *P* = 0.022) compared to saline‐treated SLE mice. These data confirm that pharmacological stimulation of the cholinergic anti‐inflammatory pathway with nicotine is capable of suppressing splenic inflammation.

### Nicotine attenuates the development of hypertension and renal injury in female mice with SLE

**Figure 3:**
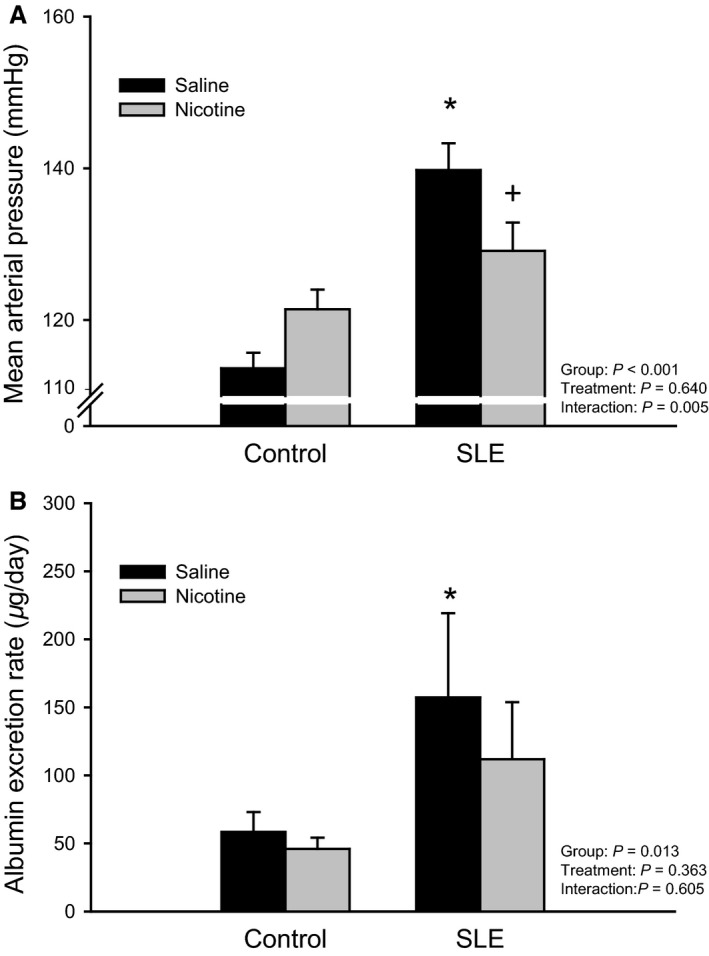
Chronic nicotine exposure attenuates the rise in blood pressure and protects from renal injury in female SLE mice: (A) Mean arterial pressure measured in control and SLE mice administered saline or nicotine for 7 days. *n *=* *7–9 animals/group; P* versus Control/Saline; *P* + versus SLE/Saline; (B) Urinary albumin excretion, an accepted index of renal injury, measured in control and SLE mice administered saline or nicotine for 7 days. *n *=* *7–12 animals/group; P* versus Control/Saline.

In order to determine whether an impaired cholinergic anti‐inflammatory pathway contributes to the pathogenesis of hypertension during SLE, mean arterial pressure was measured in mice treated with nicotine or saline. Body weight was not different between SLE animals treated with nicotine or saline throughout the study (data not shown). Mean arterial pressure was increased in SLE mice compared to controls (140 ± 4 vs. 114 ± 2 mmHg; *P* < 0.001; Fig. 3) as previously shown (Mathis et al. 2011, 2012, 2013, 2014). One week of subcutaneous nicotine therapy attenuated the rise in blood pressure in SLE mice (129 ± 4 mmHg; *P* = 0.022), but had no effect in controls (121 ± 3 mmHg).

Proteinuria as measured by Albustix was lower in SLE mice compared to controls (data not shown; grades = 1.2 ± 0.4 vs. 0.9 ± 0.3). Urinary albumin excretion rate measured by ELISA confirmed an increase in albumin in SLE mice compared to controls (157 ± 62 vs. 58 ± 15 *μ*g/day; *P* = 0.038; Fig. 3B) as previously shown (Mathis et al. 2012). Nicotine therapy blunted the rise in albumin excretion rate in SLE mice (112 ± 42).

### Nicotine blunts the accumulation of renal inflammation in female SLE mice

**Figure 4:**
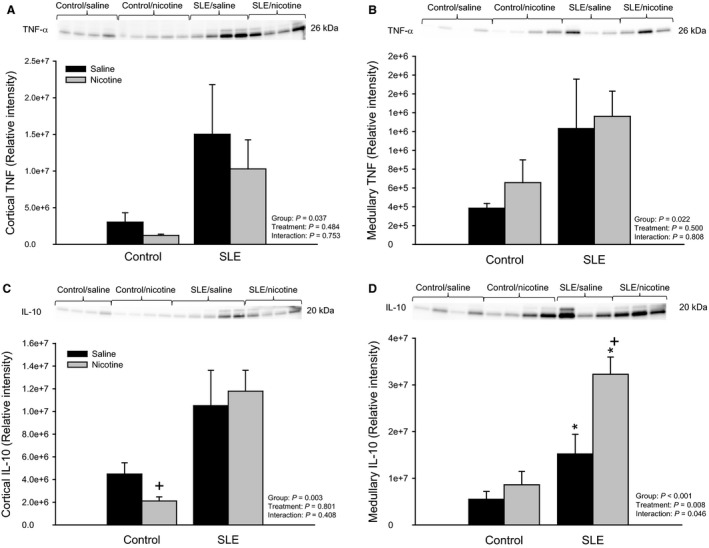
Chronic nicotine exposure alters renal inflammatory mediators in female SLE mice (A) Protein expression of TNF‐*α* assessed by Western blot in the renal cortex of control and SLE mice administered saline or nicotine for 7 days. Data are normalized to total protein. *n *=* *4–5 kidneys/group; (B) Protein expression of TNF‐*α* assessed by Western blot in the renal medulla of control and SLE mice administered saline or nicotine for 7 days. Data are normalized to total protein. *n *=* *3–4 kidneys/group; (C) Protein expression of the anti‐inflammatory cytokine, interleukin (IL)‐10, assessed by Western blot in the renal cortex of control and SLE mice administered saline or nicotine for 7 days. Data are normalized to total protein. *n *=* *4–5 kidneys/group; *P* + versus Control/Saline; (D) Protein expression of the anti‐inflammatory cytokine, interleukin (IL)‐10, assessed by Western blot in the renal medulla of control and SLE mice administered saline or nicotine for 7 days. Data are normalized to total protein. *n *=* *3–4 kidneys/group; P* versus corresponding Control; *P* + versus SLE/Saline. TNF‐*α,* tumor necrosis factor; SLE, Systemic lupus erythematosus.

There was a significant difference in mean values of renal cortical expression of TNF‐*α* between all SLE and control mice (*P* = 0.037; Fig. 4A). SLE mice treated with nicotine had 33 ± 26% less TNF‐*α* (1.0e7 ± 4.0e6) than vehicle‐treated SLE mice (1.5e7 ± 6.8e6). There was also a significant difference in mean values of renal medullary expression of TNF‐*α* between all SLE and control mice (*P* = 0.022; Fig. 4B); however, nicotine had no effect on renal medullary TNF‐*α* in SLE mice.

There was a significant difference in mean values of renal cortical expression of IL‐10 between all SLE and control mice (*P* = 0.003; Fig. 4C). Nicotine had no effect on renal cortical IL‐10 in SLE mice. Renal medullary expression of IL‐10 was increased in vehicle‐treated SLE mice compared to vehicle‐treated controls (1.5e7 ± 4.2e6 vs. 5.5e6 ± 1.7e6; *P* = 0.049; Fig. 4D). SLE mice treated with nicotine had higher medullary IL‐10 expression than vehicle‐treated SLE mice (3.2e7 ± 3.7e6; *P* = 0.004).

**Figure 5:**
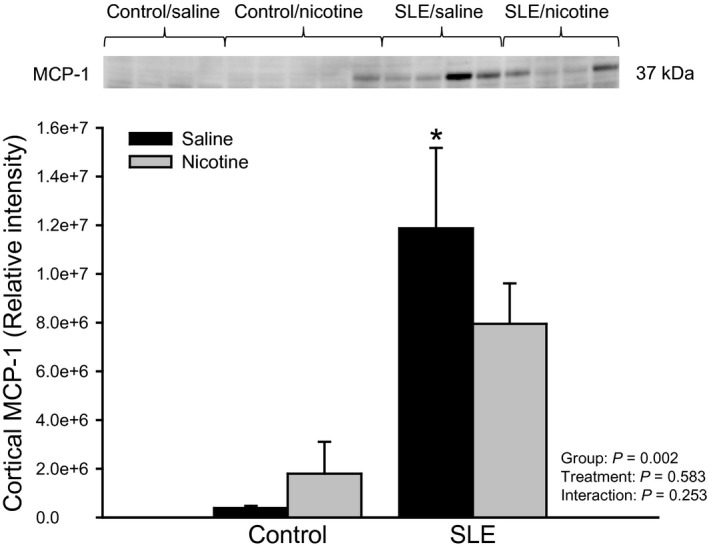
Chronic nicotine exposure alters renal cortical MCP‐1 in female SLE mice: Protein expression of dimerized MCP‐1 assessed by Western blot in kidneys of control and SLE mice administered saline or nicotine for 7 days. Data is normalized to total protein. *n *=* *4–5 kidneys/group; P* versus Control/Saline. MCP‐1, monocyte chemoattractant protein, SLE, Systemic lupus erythematosus.

Renal cortical expression of MCP‐1 was increased in vehicle‐treated SLE mice compared to vehicle‐treated controls (1.2e7 ± 3.3e6 vs. 3.9e5 ± 8.8e4; *P* = 0.002; Fig. 5). SLE mice treated with nicotine had 33 ± 14% less cortical MCP‐1 expression (8.0e6 ± 1.7e6) than vehicle‐treated SLE mice (1.2e7 ± 3.3e6); however this difference did not yield statistical significance.

### Selective agonist of the α7‐subunit of the nicotinic acetylcholine receptor prevented the development of hypertension and attenuated renal injury in SLE mice

**Figure 6:**
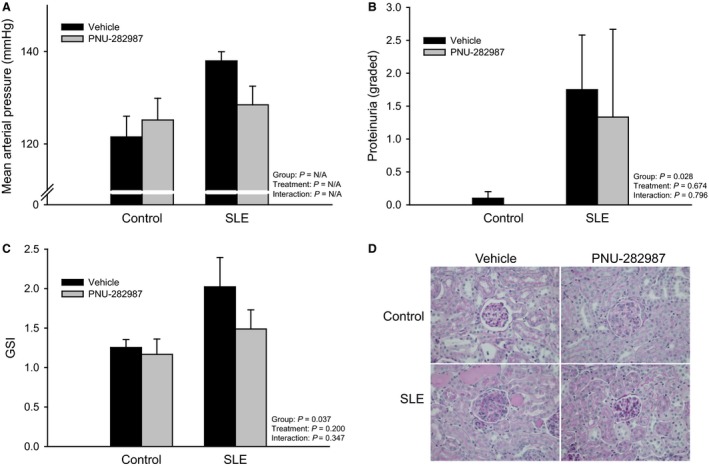
Selective cholinergic agonist (PNU‐282,987) blunts the rise in blood pressure and reduces from renal injury in female SLE mice: (A) Mean arterial pressure measured in control and SLE mice administered vehicle or PNU‐282,987 for 28 days. *n *=* *2–4 animals/group; (B) Proteinuria as measured by Albustix (“grades” calculated as described in the methods) in control and SLE mice administered vehicle or PNU‐282,987 for 28 days. *n *=* *3–5 animals/group; (C) Glomerulosclerosis index in PAS‐stained paraffin embedded kidneys of control and SLE mice administered vehicle or PNU‐292,987 for 28 consecutive days. *n *=* *3–4/group; (D) Representative pictures of PAS‐stained kidneys of control and SLE mice administered vehicle or PNU‐292,987 for 28 consecutive days. SLE, Systemic lupus erythematosus; PAS, periodic acid‐Schiff.

In order to confirm these effects are specific to peripheral actions of nicotine on the *α*7‐nAChR, we administered a selective nicotinic agonist, PNU‐282,987, or vehicle to a small subset of SLE and control mice. Splenic TNF‐*α* was increased in SLE mice compared to controls (data not shown; 0.54 ± 0.03 vs. 0.15 ± 0.03 (normalized to *β*‐actin); *P* < 0.001). In addition, mean arterial pressure was increased in SLE mice compared to controls (138 ± 2 vs. 122 ± 4 mmHg; Fig. 6A). Twenty‐eight consecutive days of intraperitoneal PNU‐282,987 reduced splenic TNF (0.33 ± 0.01; *P* = 0.002; data not shown) and prevented the rise in blood pressure in SLE mice (128 ± 4 mmHg; Fig. 6A). Renal injury in the form of albuminuria (Fig. 6B; grades = 1.8 ± 0.8 vs. 1.3 ± 1.3) and glomerulosclerosis (Fig. 6C) was lower in SLE mice treated with PNU‐282,987. There was glomerular mesangium expansion and nodular appearance of glomeruli in SLE mice and PNU‐282,987 protected from these changes (Fig. 6D).

## Discussion

The purpose of this study was to determine whether activation of the cholinergic anti‐inflammatory pathway at the level of the *α*7‐nAChR would attenuate the production and release of splenic pro‐inflammatory cytokines, which would subsequently reduce systemic, specifically renal, inflammation. We aimed to show that a reduction in renal inflammation would halt the progression of hypertension in a mouse model of SLE. To test our hypothesis, nicotine, a cholinergic agonist, was used to stimulate the pathway in hypertension‐prone SLE mice. The major findings of this study are that (1) nicotine significantly blunts splenic TNF‐*α* expression in SLE mice; (2) nicotine attenuates the development of hypertension and renal injury during SLE; and (3) nicotine suppresses renal inflammation in SLE mice. Since stimulation of the cholinergic anti‐inflammatory pathway was demonstrated to be protective in this study, these data further support the notion that this neuroimmune pathway may be impaired in SLE and strengthen the idea that chronic renal inflammation contributes to the development of hypertension.

### The cholinergic anti‐inflammatory pathway may be impaired in SLE hypertension

The inflammatory reflex provides a classic response to increases in systemic inflammation (Tracey 2002, 2009); increased pro‐inflammatory signals arrive to the central nervous system via the afferent vagus nerve and a resultant neural signal relayed by the efferent vagus nerve attenuates this inflammation. The efferent arm of this reflex, coined the ‘cholinergic anti‐inflammatory pathway’, is thought to begin with vagus nerve activation of the splenic nerve, which causes the release of acetylcholine from splenic T cells (Rosas‐Ballina et al. 2011). This acetylcholine activates cholinergic receptors, specifically the *α*7‐nAChR, and therefore inhibits the production of pro‐inflammatory cytokines such as TNF‐*α* (Wang et al. 2003; Olofsson et al. 2012). Stimulation of the cholinergic anti‐inflammatory pathway has been demonstrated to be protective in chronic inflammatory diseases (Dowling et al. 2007; Van Maanen et al. 2009; Leib et al. 2011). Hypertension is a devastating disease which has recently been linked to chronic inflammation (Guzik et al. 2007; Crowley et al. 2010; De Miguel et al. 2010; Mathis et al. 2014); however, studies testing the importance of a functional cholinergic anti‐inflammatory pathway in preventing chronic inflammation and the progression to hypertension are scarce.

SLE is a chronic autoimmune inflammatory disease that primarily affects young women in 90% of all cases (Somers et al. 2014). Given this primary patient population, the prevalence of hypertension is considerably high ranging from 40 to 74% depending on the cohort being studied (Al‐Herz et al. 2003; Sabio et al. 2011; Somers et al. 2014). This is interesting because young women are usually protected from hypertension but when placed in a setting of chronic inflammation such as lupus, the protection is lost. These characteristics alone make SLE a novel and appropriate disease model to study the link between chronic inflammation and the pathogenesis of hypertension.

Not only do SLE patients present with aberrant immune function and systemic (renal) inflammation (Budman and Steinberg 1976; Cava 2009), there is impaired autonomic function suggesting impaired vagal nerve activity. Several studies utilized autonomic function tests (e.g., cardiovascular tests such as heart rate variability, the Valsalva maneuver, deep breathing tests, heart rate response to standing) to determine the presence of autonomic dysfunction in patients with SLE and revealed significant differences when compared to normal subjects (Lagana et al. 1996; Maule et al. 1997; Louthrenoo et al. 1999; Stojanovich et al. 2007). Impaired vagal tone could contribute to a dampened cholinergic anti‐inflammatory pathway, which could partially explain the accentuated inflammation during SLE, as well as the development of renal inflammation, hypertension and renal injury. We hypothesized that the cholinergic anti‐inflammatory pathway is indeed impaired during SLE, and that stimulation of the pathway at the level of the *α*7‐nAChR would be protective against the development of hypertension. In order to test this, we utilized an established mouse model of lupus that spontaneously develops hypertension. We have found that renal inflammation (e.g., increased renal cortical expression of TNF‐*α* and MCP‐1) and renal injury (e.g., glomerulosclerosis) are associated with the hypertension in these female SLE mice (Mathis et al. 2013, 2014).

### Subcutaneous nicotine stimulates the cholinergic anti‐inflammatory pathway in female SLE mice

In this study, nicotine was chosen to pharmacologically stimulate the cholinergic anti‐inflammatory pathway at the level of the *α*7‐nAChR in control and SLE animals. Nicotine and other cholinergic agonists have been shown in previous studies to significantly reduce the production of pro‐inflammatory mediators (e.g., TNF‐*α*, Th1 and Th17 cells) through this receptor in models of ischemia‐reperfusion injury (Sadis et al. 2007; Yeboah et al. 2008), but also in experimental models of sepsis and pregnancy‐induced hypertension (Wang et al. 2004; Dowling et al. 2007). Other studies provided evidence that stimulation of the cholinergic anti‐inflammatory pathway with nicotine requires an intact *α*7‐nAChR to suppress an immune response and subsequently attenuate inflammation (Nizri et al. 2009). Following 7‐day subcutaneous nicotine exposure in the current study, we observed a decrease in TNF‐*α* produced in the spleen of SLE mice. This serves as evidence that the cholinergic anti‐inflammatory pathway can be stimulated with peripheral administration of nicotine and confirms that such activation of the *α*7‐nAChR suppresses inflammation.

### Stimulation of splenic cholinergic receptors protect from the development of hypertension and attenuated renal injury in female SLE mice

With confirmation of an activated cholinergic anti‐inflammatory pathway in SLE mice following chronic nicotine administration, we were then able to probe whether blood pressure was affected. While *acute* nicotine is typically thought to cause a hypertensive response (Armitage 1965; Haass and Kubler 1996; Fox et al. 2012), we found that *chronic* nicotine exposure reduced blood pressure in SLE mice. We confirmed the importance of stimulating the splenic *α*7‐nAChR in this protection by administering a different, yet selective, agonist of the receptor and saw the same protection against hypertension in SLE mice. One mechanism that may be involved in the protective attributes of nicotine and its ability to activate the cholinergic anti‐inflammatory pathway in SLE mice may be the reduction in renal inflammation. Previous studies have linked TNF‐*α* mechanistically to SLE hypertension. Renal inflammation may lead to alterations in the renal vasculature, which would therefore alter renal hemodynamics and shift the pressure natriuresis relationship toward hypertension. (Venegas‐Pont et al. (2010) demonstrated that pharmacological blockade of TNF‐*α* via etanercept prevented hypertension in SLE mice. In the current study, we saw slight reductions in renal cortical TNF‐*α* and MCP‐1 expression following nicotine administration. In addition to lowering renal inflammatory mediators, nicotine blunted the level of injury in the kidneys of SLE mice, as indicated by urinary albumin excretion. These findings complement other studies that have shown a protective effect of nicotine on renal injury (Sadis et al. 2007; Yeboah et al. 2008; Agarwal et al. 2012). The dose of nicotine administered in the current study (2 mg/kg) has been shown to significantly reduce inflammation when administered for 28 consecutive days in female C57 mice induced with experimental autoimmune encephalomyelitis (Nizri et al. 2009). The modest changes in inflammation following nicotine administration in female SLE mice may be explained by the shorter duration of nicotine treatment. Taken together, these data suggest that in a setting of chronic inflammation, nicotine may activate the cholinergic anti‐inflammatory pathway and help suppress the release of inflammatory mediators from the spleen, culminating in reduced systemic (renal) inflammation.

The results of our study provide additional evidence that the cholinergic anti‐inflammatory pathway is impaired in SLE mice. Further studies are needed to investigate the role of an intact cholinergic anti‐inflammatory pathway in preventing the development of chronic inflammation. It is of interest to determine whether stimulation of the cholinergic anti‐inflammatory pathway, pharmacologically at the level of cholinergic receptors or upstream at the level of the vagus nerve, is indeed beneficial during hypertension in the setting of chronic inflammation. The latter would be of significant clinical relevance given the recent advances in implantable vagus nerve stimulators in both humans and animals (Multon and Schoenen 2005; Van Leusden et al. 2015).

### Significance

SLE is an autoimmune disorder that predominantly affects women of child‐bearing age. Immune and inflammatory dysregulation, autonomic dysfunction, and hypertension are all manifestations of SLE that qualify SLE as a suitable disease model to investigate the relationship between neuroimmune mechanisms and the development of hypertension. Recent studies have demonstrated the role of chronic inflammation in the pathogenesis of hypertension; however, the role of the “cholinergic anti‐inflammatory pathway” in the development of this inflammation has not been established (Li et al. 2011; Chen et al. 2012). Future studies seek to explore the relationship of such neuroimmune pathways in the pathogenesis of SLE hypertension, as well as essential hypertension, in hopes to lead to the advancement of novel therapeutic targets.

## Conflict of Interest

None declared.
